# Impact of menopause and age on breast density and background parenchymal enhancement in dynamic contrast-enhanced magnetic resonance imaging

**DOI:** 10.1117/1.JMI.12.S2.S22002

**Published:** 2025-03-11

**Authors:** Grey Kuling, Jennifer D. Brooks, Belinda Curpen, Ellen Warner, Anne L. Martel

**Affiliations:** a University of Toronto, Department of Medical Biophysics, Toronto, Ontario, Canada; b Blavatnik Institute, Harvard Medical School, Department of Biomedical Informatics, Boston, Massachusetts, United States; c University of Toronto, Dalla Lana School of Public Health, Toronto, Ontario, Canada; d Sunnybrook Health Sciences Centre, Division of Breast Imaging, Toronto, Ontario, Canada; e University of Toronto, Department of Medical Imaging, Toronto, Ontario, Canada; f Sunnybrook Odette Cancer Centre, Division of Medical Oncology and Hematology, Toronto, Ontario, Canada; g University of Toronto, Department of Medicine, Toronto, Ontario, Canada; h Sunnybrook Research Institute, Physical Sciences, Toronto, Ontario, Canada

**Keywords:** breast density, background parenchymal enhancement, dynamic contrast-enhanced magnetic resonance imaging, menopause, imaging biomarkers, breast cancer risk

## Abstract

**Purpose:**

Breast density (BD) and background parenchymal enhancement (BPE) are important imaging biomarkers for breast cancer (BC) risk. We aim to evaluate longitudinal changes in quantitative BD and BPE in high-risk women undergoing dynamic contrast-enhanced magnetic resonance imaging (DCE-MRI), focusing on the effects of age and transition into menopause.

**Approach:**

A retrospective cohort study analyzed 834 high-risk women undergoing breast DCE-MRI for screening between 2005 and 2020. Quantitative BD and BPE were derived using deep-learning segmentation. Linear mixed-effects models assessed longitudinal changes and the effects of age, menopausal status, weeks since the last menstrual period (LMP-wks), body mass index (BMI), and hormone replacement therapy (HRT) on these imaging biomarkers.

**Results:**

BD decreased with age across all menopausal stages, whereas BPE declined with age in postmenopausal women but remained stable in premenopausal women. HRT elevated BPE in postmenopausal women. Perimenopausal women exhibited decreases in both BD and BPE during the menopausal transition, though cross-sectional age at menopause had no significant effect on either measure. Fibroglandular tissue was positively associated with BPE in perimenopausal women.

**Conclusions:**

We highlight the dynamic impact of menopause on BD and BPE and correlate well with the known relationship between risk and age at menopause. These findings advance the understanding of imaging biomarkers in high-risk populations and may contribute to the development of improved risk assessment leading to personalized chemoprevention and BC screening recommendations.

## 1 Introduction

Stratified breast cancer (BC) screening strategies play a critical role in providing personalized and cost-effective screening for high-risk patients. Incorporating breast magnetic resonance imaging (MRI) alongside mammography for high-risk individuals, based predominantly on family history and/or genetic status, has been observed to enhance sensitivity in cancer detection.^1^^–^^4^ However, a more accurate estimation of individual risk would facilitate decisions regarding chemoprevention, risk-reducing surgery, screening strategies for high-risk patients, and optimizing MRI techniques and frequency.

Factors such as breast density (BD) and background parenchymal enhancement (BPE), as measured in dynamic contrast-enhanced magnetic resonance imaging (DCE-MRI), have been found to modify cancer risk.^5^^–^^8^ These factors should be considered for BC screening risk stratification and clinical decision-making. Despite the common use of subjective categories from BI-RADS^9^ for BD and BPE, quantitative measures have been found to be better predictors of risk.^10^^–^^17^ Although covariates such as age, body mass index (BMI), weeks since last menstrual period (LMP-wks), and hormone replacement therapy (HRT) influence the BD and BPE BI-RADS category,^18^^–^^20^ their impact on quantitative measurements remains unexplored.

There is a notable absence of longitudinal analyses demonstrating how these quantitative measurements change as women go through premenopause, perimenopause, and postmenopause. Our retrospective observational study assesses this using linear mixed-effects models because of their robust and established applications in repeated measures in longitudinal studies.^21^^–^^23^ The analysis controls for variables such as the LMP-wks, BMI, and HRT when analyzing longitudinal repeat measures of BD and BPE obtained from a high-risk cohort undergoing annual MRI screening. Our study utilizes linear mixed-effects models to accurately estimate population BD and BPE changes across menopause and its stages in women at high risk for BC.

## 2 Methods

### 2.1 Sample

The ethics board at Sunnybrook Research Institute approved a retrospective observational study that collected breast MRIs from high-risk women undergoing breast screening at Sunnybrook Health Sciences Centre (SHSC) between 2005 and 2020. Patient consent was waived.

In Ontario, Canada, women at high risk for BC are eligible to receive government-funded annual mammography and breast MRI screening between ages 30 and 69.^24^ High-risk criteria include a BRCA1/2 germline mutation, being a first-degree relative of a mutation carrier, a history of significant radiation exposure (such as radiation therapy for lymphoma before age 30), or an IBIS/BOADICEA BC risk score exceeding 25%. The SHSC database contains over 10,000 breast MRI exams from 2384 women,^25^ of whom 2296 (96.3%) had medical records at SHSC for more than 1.5 years. Of those, 1298 (56.5%) had no personal history of BC before their first MRI exam, as indicated by BI-RADS BERT,^26^ a specialized large language model for breast imaging. Among these 1298 patients, 1039 (80.0%) did not develop BC during their captured medical records at SHSC (See Table 1).

**Table 1:** Patient inclusion criteria.

Requirement		Patient count		
Total amount of patients		2384		
Patient record >1.5 years		2296		
No history of breast cancer before first MRI exam		1298		
Did not develop breast cancer during patient record		1039		
Menopausal groupings		Premenopausal	Perimenopausal	Postmenopausal
		561	145	333
Change in menopausal status (±3 years from program entry/exit)		N/A	99	N/A
Patients with valid DCE-MRI data and segmentation	BD	476	89	269
	BPE	457	89	258

### 2.2 Measures: Menopausal Status

Menopausal status at the time of imaging was determined by recording their last menstrual period from the clinical history/indication sections of the radiology report. Premenopausal status was defined as having a menstrual period within 12 months of the exam, mention of recent pregnancy, use of intra-uterine devices or oral contraceptives, or a statement of “irregularity.” The patient was considered postmenopausal if the radiologist had recorded an oophorectomy (with or without hysterectomy), postmenopausal, post, none, or N/A for the LMP-wks. All other menopausal statuses were assigned to a “Not Stated” label. If the menopausal status was not stated in the report, the most recent (9 to 15 months) exam’s menopausal status was assumed to still stand.

The 1039 women with menopausal status recorded patients were divided into premenopausal, perimenopausal, and postmenopausal groups during their screening history in our database. Of the 1039 women, 561 patients were consistently recorded as premenopausal at their breast MRI exam times, whereas 333 had consistent postmenopausal exams. In the patient history report, it was found that 145 patients had gone through menopause during their screening study period. Ninety-nine patients had a change in menopausal status 3 years before exiting/after entering the screening program. By setting lower and upper boundaries for specific exams to measure BD and BPE, we can ensure an accurate assessment of quantitative changes before and after menopause (see Table 1). These 99 patients were labeled as the perimenopausal group. This classification of the perimenopausal group may differ from previous publications or clinical usage; however, it was deemed the most suitable designation for our study aimed at examining the impact of menopause on BD and BPE.

### 2.3 Measures: Quantitative Tissue Factors

Quantitative measures of BD and BPE were calculated from breast MRI scans using deep learning segmentation^17^ applied to pre-contrast images to isolate the fat and fibroglandular tissue (FGT) components of the breast. Each MRI exam consists of a bilateral T1-weighted (T1w) scan acquired on a 1.5-T magnet (Signa, GE) using a dedicated breast coil in a sagittal orientation for higher spatial resolution, faster acquisition, and better alignment with the mammographic mediolateral oblique view. Scans have 0.39×0.39  mm in-plane resolution and 3.0-mm slice thickness and include pre- and post-contrast fat-suppressed T1w images.

We evaluated the absolute volume of FGT (|FGT|; units: ${\mathrm{cm}}^{3}$) and the percent of FGT (%FGT; units:%) for BD measurements from the segmentation maps. For BPE, we evaluated the enhancement percentage in a signal enhancement ratio map derived from the first post-contrast timepoint of DCE-MRI (around 160 s on average). This aligns with the BI-RADS lexicon^9^ recommendation for optimal lesion enhancement and BPE assessment. Pre-contrast FGT segmentation maps were applied to post-contrast images with timepoint registration using optical flow image registration.^27^ We quantified BPE as percent BPE (%BPE; units: %)^28^ as the percentage of enhancing FGT with a signal enhancement ratio exceeding 0.2. We calculated the percent difference in mean parenchymal enhancement within the FGT segmentation (MPE; units: %) between pre- and post-contrast images.

### 2.4 Measures: Covariates

Our analysis considered the impact of HRT, BMI, and LMP-wks on both BD and BPE. We recorded HRT as a binary variable from the radiology report if it mentioned any HRT or HRT medication/intervention for postmenopausal patients, including Angeliq, EstroGel, Premarin, and Estriol. This was reassessed with each round of breast MRI screening exam. Our records did not differentiate between types of HRT, such as estrogen-alone HRT versus combined estrogen/progestin HRT. As a result, we were unable to account for potential differences in HRT type among patients.

BMI was estimated using the total amount of fat in the breast (|Fat|; units: ${\mathrm{cm}}^{3}$) as this strongly correlates with the patient’s BMI.^5^ The |Fat| was measured by voxel count times the voxel volume. For BPE measures, regardless of menopausal status, we also adjusted for the influence of |FGT| on the measurements by taking the total volume of FGT as an independent covariate.

For the premenopausal women, we found the LMP-wks and quantified it as the number of weeks since the last period. Patients are usually advised to schedule a scan during week 2 of their menstrual cycle, but they are not denied a scan if they report a different week. This amount was further categorized into ordinal categories of LMP-wks < 1 week, 1 < LMP-wks < 2 weeks, 2 < LMP-wks < 3 weeks, 3 < LMP-wks < 4 weeks, and 4 <LMP-wks < 26 weeks. These bins were used to account for any irregular menstruation.

### 2.6 Statistical Analysis

We performed LME modeling with random intercepts. Random intercepts were chosen to improve the mixed effects model’s flexibility and determine the population’s rate of change over time while accounting for individual variations from the population intercept baseline. We used a linear mixed effect modeling technique developed and validated by Morell, Brant, and Ferrucci,^22^ where age is split into two variables: age at the first measurement (FAge) and years since the first measurement (YrSncFAge). This has been shown to have a closer fit to data, which would give a more accurate description of factor trends over time than using age alone. The mathematical expressions for this are

$$\begin{array}{l} \text{Measurement}∼\text{Age}+(1|\text{Patient})+\text{Covariates}\ldots \\ \text{Measurement}∼\text{FAge}+\mathrm{YrSncFAge}+(1|\text{Patient})+\text{Covariates}\ldots \end{array}$$

This model was fit for the pre- and post-menopausal groups. This split separates age at the time of measurement into a cross-sectional and longitudinal portion, providing a better understanding of how aging affects the measurements. Each patient was treated as a cluster of measurements in the mixed effects. Therefore, quantitative measurements of a given exam were averaged across the left and right breasts and different scan acquisitions in the exam if they were available.

For the perimenopausal group, we took inspiration from Morell, Brant, and Ferrucci.^22^ We analyzed the mixed effects on the cross-sectional age at menopause (MAge) and the longitudinal years since menopause (YrSncMAge).

Measurement∼MAge+YrSncMAge+(1|Patient)+Covariates…

This could give a more aligned interpretation of how significant the rate of change is across menopause and reveal the effect of age at menopause on the response variable separately. The date of menopause was estimated to be halfway between the two scans, where the patient’s record changes from premenopausal to postmenopausal based on the labeling in the prior subsection. Following this estimation, the patient’s YrSncMAge premenopausal value becomes negative, whereas the postmenopausal value becomes positive.

In our analysis, certain exams were excluded due to computational issues with segmentation (Table 1). Specifically, 85 premenopausal, 10 perimenopausal, and 64 postmenopausal patients were excluded due to voxel resolution mismatches and severe time registration issues that could not be corrected using registration algorithms. In addition, exams were omitted due to missing DCE-MRI data, excluding 19 patients from the premenopausal group and 11 patients from the postmenopausal group; all patients were included in the perimenopausal group. In our study, P-values lower than 0.05 were considered statistically significant.

## 3 Results

### 3.1 Premenopausal Group

The findings of our experiment have revealed noteworthy longitudinal trends and confounding factors affecting %FGT and |FGT| in premenopausal women (Table 2). Our analysis did not yield statistical significance for the age at first measurement (%FGT: −0.12%/year, p=0.065; $|\mathrm{FGT}|:-0.54\;{\mathrm{cm}}^{3}/\mathrm{year}$, p=0.336). However, it did demonstrate longitudinal effects on %FGT and |FGT|, with %FGT declining by 0.3% annually ($p=3.4\times 10^{-4}$) and |FGT| decreasing by $1.79\;{\mathrm{cm}}^{3}$ per year (p=0.001). Both measures were influenced by |Fat|, a surrogate for BMI, with |Fat| decreasing %FGT by $0.01\%\;\mathrm{per}\;{\mathrm{cm}}^{3}$ ($p=1.5\times 10^{-79}$) and increasing |FGT| by $0.01\;{\mathrm{cm}}_{\mathrm{FGT}}^{3}/{\mathrm{cm}}_{\text{Fat}}^{3}$ (p=0.023). No statistical impact was observed for weeks since the last menstrual period (%FGT: 0.20%/wk., p=0.077; $|\mathrm{FGT}|:1.20\;{\mathrm{cm}}^{3}/\mathrm{wk}.$, p=0.110).

**Table 2:** Relationship between age and BD and BPE in premenopausal women adjusting for |Fat|, LMP-wks, and |FGT| using linear mixed-effects models.

Breast density						
No. of measurements: 1024, no. of patients: 476, min group size: 1, max group size: 6, mean group size: 2.2						
Variables	%FGT			|FGT|		
	Coef.	95% CI	p-value	Coef.	95% CI	p-value
FAge	−0.12	[−0.25, 0.01]	0.07	−0.54	[−1.65, 0.56]	0.34
YrsSncFAge	−0.30	[−0.58, −0.24]	$3.41\times 10^{-4}$	−1.79	[−2.86, −0.72]	$1.02\times 10^{-3}$
Intercept	28.26	[22.85, 33.68]	$1.41\times 10^{-24}$	117.47	[71.46, 163.48]	$5.62\times 10^{-7}$
|Fat|	−0.01	$\left[-0.01,-9.0\times 10^{-3}\right]$	$1.49\times 10^{-79}$	0.01	$\left[1.0\times 10^{-3},0.02\right]$	0.02
LMP-wks	0.20	[−0.02, 0.43]	0.08	1.20	[−0.27, 2.67]	0.11
Background parenchymal enhancement						
No. of measurements: 927, no. of patients: 457, min group size: 1, max group size: 6, mean group size: 2.0						
Variables	%BPE			MPE		
	Coef.	95% CI	*p*-value	Coef.	95% CI	*p*-value
FAge	−0.03	[−0.34, 0.28]	0.851	−0.05	[−0.27, 0.216]	0.643
YrsSncFAge	−0.46	[−1.41, 0.50]	0.347	−0.69	[−1.39, 0.02]	0.057
Intercept	49.14	[35.76, 62.53]	$6.22\times 10^{-13}$	27.05	[17.78, 36.33]	$1.08\times 10^{-8}$
|Fat|	$3.0\times 10^{-3}$	$\left[1.0\times 10^{-4},6.0\times 10^{-3}\right]$	0.04	$1.2\times 10^{-3}$	$\left[-1.0\times 10^{-3},3.0\times 10^{-3}\right]$	0.237
|FGT|	0.01	[−0.02, 0.03]	0.621	0.01	$\left[-4.0\times 10^{-3},0.03\right]$	0.121
LMP-wks	−1.54	[−2.84, −0.25]	0.02	−0.90	[−1.86, 0.05]	0.063

Furthermore, %BPE showed no age-related trend cross-sectionally or longitudinally and did not exhibit an impact from |FGT|. However, %BPE was influenced by |Fat| at a rate of $3.0\times 10^{-3}\%\;\mathrm{per}\;{\mathrm{cm}}^{3}$ (p=0.049), and the LMP-wks was found to decrease %BPE by 1.54%/wk (p=0.020).

### 3.2 Postmenopausal Group

In our investigation involving postmenopausal women (Table 3), we observed notable age-related declines in %FGT and |FGT|, with both metrics exhibiting cross-sectional decreases over time. Specifically, %FGT demonstrated a decrease of 0.13% annually (p=0.008), whereas |FGT| decreased by $1.16\;{\mathrm{cm}}^{3}$ annually (p=0.017). Longitudinally, %FGT displayed an annual decrease of 0.38% per year ($p=3.5\times 10^{-10}$), and |FGT| decreased by $2.75\;{\mathrm{cm}}^{3}$ per year ($p=5.1\times 10^{-10}$). We found a statistical impact of |Fat| on %FGT that decreases $4.7\times 10^{-3}\%/{\mathrm{cm}}^{3}$ ($p=6.8\times 10^{-32}$), but no impact on |FGT|. Notably, we did not identify any statistically significant impact of HRT on %FGT and |FGT| in postmenopausal women.

**Table 3:** Relationship between age and BD and BPE in postmenopausal women adjusting for |Fat|, HRT, and |FGT| using linear mixed-effects models.

Breast density						
No. of measurements: 692, no. of patients: 269, min group size: 1, max group size: 7, mean group size: 2.6						
Variables	%FGT			|FGT|		
	Coef.	95% CI	p-value	Coef.	95% CI	p-value
FAge	−0.13	[−0.23, −0.04]	$8.04\times 10^{-3}$	−1.16	[−2.12, −0.21]	0.02
YrsSncFAge	−0.38	[−0.37, −0.19]	$3.48\times 10^{-10}$	−2.75	[−3.62, −1.88]	$5.09\times 10^{-10}$
Intercept	19.85	[14.41, 25.29]	$8.23\times 10^{13}$	126.66	[73.92, 179.41]	$2.52\times 10^{-6}$
|Fat|	$-4.7\times 10^{-3}$	$\left[-6.0\times 10^{-3},-4.0\times 10^{-3}\right]$	$6.77\times 10^{-32}$	$4.6\times 10^{-3}$	$\left[-3.0\times 10^{-3},0.01\right]$	0.252
HRT	0.63	[−0.10, 1.37]	0.090	3.89	[−3.39, 11.18]	0.295
Background parenchymal enhancement						
No. of measurements: 623, no. of patients: 258, min group size: 1, max group size: 6, mean group size: 2.4						
Variables	%BPE			MPE		
	Coef.	95% CI	*p*-value	Coef.	95% CI	*p*-value
FAge	−0.35	[−0.62, −0.08]	0.012	−0.22	[−0.38, −0.06]	0.006
YrsSncFAge	−1.11	[−1.91, −0.30]	0.007	−0.57	[−1.01, −0.12]	0.013
Intercept	50.66	[35.03, 66.29]	$2.12\times 10^{-10}$	25.94	[16.89, 35.0]	$1.96\times 10^{-8}$
|Fat|	0.01	$\left[5.0\times 10^{-3},1.2\times 10^{-2}\right]$	$3.99\times 10^{-6}$	$4.6\times 10^{-3}$	$\left[3.0\times 10^{-3},7.0\times 10^{-3}\right]$	$7.40\times 10^{-6}$
|FGT|	−0.04	$\left[-0.07,-3.0\times 10^{-3}\right]$	0.033	$-3.3\times 10^{-3}$	[−0.02, 0.02]	0.725
HRT	7.01	[0.22, 13.79]	0.043	2.77	[−0.99, 6.54]	0.149

Regarding BPE, a significant decline with cross-sectional age was observed, with %BPE decreasing by 0.35% annually (p=0.012) and MPE decreasing by 0.22% per year (p=0.006). Longitudinally, %BPE exhibited a reduction of 1.11% per year (p=0.007), whereas MPE decreased by 0.57% annually (P=0.013). |Fat| was found to significantly affect both measures of BPE, with |Fat| increasing %BPE by $0.01\%\;\mathrm{per}\;{\mathrm{cm}}^{3}$ ($P=4.0\times 10^{-6}$) and MPE by $4.6\times 10^{-3}\%\;\mathrm{per}\;{\mathrm{cm}}^{3}$ ($p=7.4\times 10^{-6}$). Our analysis did not reveal a significant impact on MPE from |FGT| or HRT. However, we observed that |FGT| reduced %BPE by $0.04\%\;\mathrm{per}\;{\mathrm{cm}}^{3}$ (p=0.033), and HRT increased %BPE by 7.01% (p=0.043).

### 3.3 Perimenopausal Group

In our investigation involving perimenopausal women (Table 4), we observed that the cross-sectional age at menopause exhibited no significant influence on %FGT or |FGT|. However, the longitudinal years in a woman’s transition from premenopausal to postmenopausal demonstrated a notable impact on both FGT measures. Specifically, %FGT exhibited a reduction of 1.13% per year ($p=3.2\times 10^{-19}$) as women progressed from premenopausal to postmenopausal status, whereas |FGT| decreased by $10.19\;{\mathrm{cm}}^{3}$ annually during this transition ($p=2.8\times 10^{-19}$). Our findings revealed that |Fat| emerged as a significant factor solely for %FGT, contributing to a decrement of $8.8\times 10^{-3}\%\;\mathrm{per}\;{\mathrm{cm}}^{3}$ ($p=1.6\times 10^{-11}$).

**Table 4:** Relationship between age and BD and BPE in perimenopausal women adjusting for |Fat| and |FGT| using linear mixed-effects models.

Breast density						
No. of measurements: 258, no. of patients: 89, min group size: 1, max group size: 6, mean group size: 2.9						
Variables	%FGT			|FGT|		
	Coef.	95% CI	p-value	Coef.	95% CI	p-value
MAge	$-5.0\times 10^{-3}$	[−0.35, 0.34]	0.977	0.29	[−2.27, 2.85]	0.826
YrsSncMAge	−1.13	[−1.43, −0.823]	$3.19\times 10^{-13}$	−10.19	[−12.41, −7.96]	$2.79\times 10^{-19}$
Intercept	21.83	[5.54, 38.11]	0.009	77.46	[−44.03, 198.95]	0.211
|Fat|	$-8.8\times 10^{-3}$	$\left[-0.01,-6.0\times 10^{-3}\right]$	$1.62\times 10^{-11}$	$1.2\times 10^{-2}$	[−0.01, 0.03]	0.201
Background parenchymal enhancement						
No. of measurements: 240, no. of patients: 89, min group size: 1, max group size: 6, mean group size: 2.7						
Variables	*%*BPE			MPE		
	Coef.	95% CI	*p*-value	Coef.	95% CI	*p*-value
MAge	−0.50	[−1.08, 0.11]	0.113	−0.32	[−0.74, 0.10]	0.132
YrsSncMAge	−2.34	[−3.98, −0.70]	0.005	−1.24	[−2.46, −0.02]	0.047
Intercept	50.06	[20.59, 79.52]	0.001	29.34	[8.85, 49.83]	0.005
|Fat|	$1.6\times 10^{-3}$	$\left[-5.0\times 10^{-3},8.0\times 10^{-3}\right]$	0.628	$-3.5\times 10^{-4}$	$\left[-5.0\times 10^{-3},4.0\times 10^{-3}\right]$	0.878
|FGT|	0.08	[0.03, 0.13]	0.008	0.05	[0.01, 0.08]	0.009

Regarding BPE, we did not observe a statistically significant association concerning the cross-sectional age of menopause. However, during the transition from premenopausal to postmenopausal status, we documented substantial declines in both %BPE and MPE. Specifically, %BPE exhibited a decrease of 2.34% per year (p=0.005), whereas MPE decreased by 1.24% annually (p=0.047). Notably, these measures were independent of |Fat| during this transition. Nevertheless, |FGT| emerged as a statistically significant factor, showing an increase of 0.08% in %BPE $\mathrm{per}\;{\mathrm{cm}}^{3}$ of |FGT| (p=0.008), along with a 0.05% increase in MPE $\mathrm{per}\;{\mathrm{cm}}^{3}$ of |FGT| (p=0.009).

## 4 Discussion

This study delves into the nuanced interplay between menopause and various factors influencing breast composition measured through DCE-MRI. In premenopausal women, age and BMI affect BD measurements, whereas %BPE is driven by the timing of the scan in the menstrual cycle and BMI. The transition to menopause marks a period of a significant decrease in both BD and BPE quantitative measurements. Among postmenopausal women, longitudinal changes in cross-sectional and longitudinal age affect BD, whereas BMI and HRT additionally influence %BPE.

Our findings align with prior studies demonstrating a qualitative decrease in BD and BPE as women transition from premenopausal to postmenopausal. Brooks et al.^29^ observed this pattern with categorical BPE ranking, and our results using mixed-effects models provide further validation. Their study found a hormonal influence on %BPE but not MPE. LMP-wks had a negative association with %BPE, indicating a decrease in %BPE further from a woman’s LMP-wks, contrary to historical observations of radiologists.^19^ However, limitations in our mixed-effects modeling, which only included mixed intercepts and not slopes, may explain this discrepancy. Including LMP-wks as a mixed slope in future studies could reveal a population trend differing from the observed negative association.

Additional limitations of this study include its single-institution data source and potential selection bias from unreliable natural language processing algorithms. The analyzed radiology reports may have underreported certain variables, such as oral contraceptive use and BRCA status, and did not differentiate between estrogen-alone and combined estrogen/progestin HRT, limiting our ability to assess potential differences in imaging outcomes. Using sagittal protocols for breast DCE-MRI may introduce measurement bias. Splitting data into menopausal status groups and only including patients without BC lowered our sample size, thereby reducing the statistical robustness of our findings. Additional data from other sources are needed to verify the models developed.

The findings provide insights into the factors that impact BD and BPE. Risk analysis should include these factors as covariates for a more comprehensive understanding. Although some smaller studies with limited covariate adjustment report no correlation between BPE and BC risk,^30^ larger and more comprehensive analyses do observe a significant association, underscoring the importance of rigorous study designs to clarify BPE’s predictive value.^5^ Our results also show that BD and BPE significantly change as women enter menopause; consequently, menopausal status needs to be considered for any future analysis of the effects of these factors on BC risk. An illustrative clinical scenario could involve two 45-year-old women, patient A and patient B, with similar high-risk profiles, BD and BPE. Over the subsequent 5 years, patient A enters menopause, whereas patient B does not. As a result, BD and BPE decreased significantly for patient A compared with patient B. This hypothetical scenario suggests that patient B, due to the elevated BD and BPE, may be more inclined to consider tamoxifen for chemoprevention^31^ than patient A, whose decreased BD and BPE may indicate a lower risk. Consequently, it raises the prospect of recommending a risk reassessment every 5 years between ages 40 and 60 to account for the dynamic impact of menopausal status on BC risk assessment.

Our study may offer a new approach to BC risk assessment for postmenopausal patients. Although BD and BPE tend to decrease with age, overall BC risk increases the rate of decline in BD, and BPE across the transition into menopause in an individual woman may offer a more precise and personalized assessment of BC risk than static measurements.

In addition, this study provides insights into the impact of HRT on BD and BPE. The effect of HRT on risk is complex and depends on factors such as patient age, type of HRT, and duration of use.^32^ As we have not accounted for all of these variables, we can only conclude that HRT has an average effect on BD and BPE. Future studies will be necessary to determine whether the increase in BPE is clinically significant. If it is, changes in BPE could be used to identify which patients should discontinue HRT and which can safely continue.

Furthermore, recent studies have found a link between baseline oestradiol serum levels and the effectiveness of using the aromatase inhibitor anastrozole to prevent BC in postmenopausal women.^33^ BD and BPE have also been found to be associated with the response to hormonal therapies for estrogen receptor-positive tumors.^34^^,^^35^ Persistently high BPE or BD measurements, even after menopause, may help identify women most likely to benefit from chemoprevention with aromatase inhibitors.

## 5 Conclusion

This study reveals insights into the effects of age, menstrual status, and other covariates on DCE-MRI quantitative BD and BPE, potentially leading to improved risk assessment and more personalized prevention and screening strategies for BC. Further exploration is needed to develop personalized approaches for BC screening, considering the dynamic and multifaceted nature of breast composition throughout a woman’s life.

## Data Availability

The datasets generated and/or analyzed during this study are not publicly available due to the lack of ethics approval for data sharing and the significant effort required for anonymization and regulatory compliance. However, data may be made available upon reasonable request to the corresponding author, subject to institutional and ethical review board approval. The code used for this study is not publicly accessible but may be provided to qualified researchers upon reasonable request to the corresponding author. No physical materials were used in this study.
